# Supplementary material for: Split-pulse X-ray photon correlation spectroscopy with seeded X-rays from X-ray laser to study atomic-level dynamics
Source: Nat Commun. 2020 Dec 4;11:6213. doi: 10.1038/s41467-020-20036-z (PMC7718898; doi:10.1038/s41467-020-20036-z)
Supplement: Supplementary file 1 — Supplementary Information [file 41467_2020_20036_MOESM1_ESM.pdf]

SUPPLEMENTARY INFORMATION

Split-pulse X-ray photon correlation spectroscopy with seeded X-rays from X-ray laser to study atomic-level dynamics

Yuya Shinohara[1*], Taito Osaka[2], Ichiro Inoue[2], Takuya Iwashita[3], Wojciech Dmowski[4], Chae Woo Ryu[4], Yadu Sarathchandran[5], Takeshi Egami[1,4,5]

[1]*Materials Science and Technology Division, Oak Ridge National Laboratory, Oak Ridge, Tennessee 37831, USA*

[2]*RIKEN SPring-8 Center, Sayo, Hyogo 679-5198, Japan*

[3]*Department of Integrated Science and Technology, Oita University, Dannoharu, Oita 870-1192, Japan*

[4]*Department of Materials Science and Engineering, The University of Tennessee, Knoxville, Tennessee, 37996 USA*

[5]*Department of Physics and Astronomy, The University of Tennessee, Knoxville, Tennessee, 37996 USA*

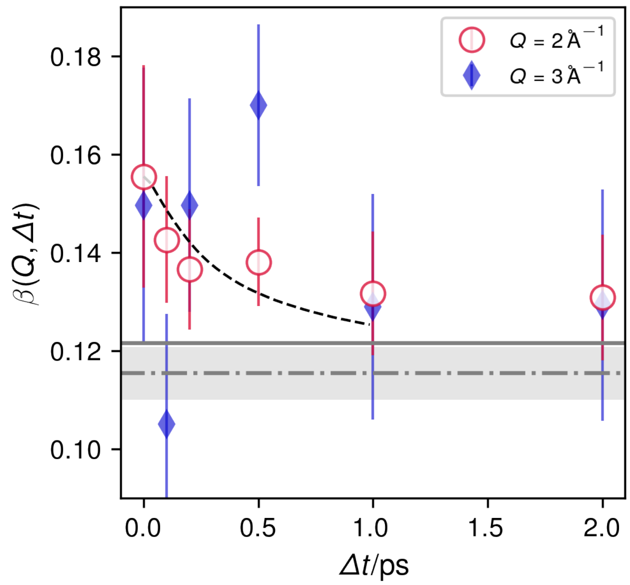

Supplementary Figure S1. X-ray speckle contrast obtained by maximum-likelihood estimation with $I_{\mathrm{fixed}} < 3$ μJ. The solid line ($Q = 2.00 \pm 0.06$ Å^{-1}) and dot-dashed line ($Q = 3.00 \pm 0.04$ Å^{-1}) represent the contrast measured when there was no overlap between two sub-pulses. The uncertainty was calculated using the second derivative of the log-likelihood,[20] and the uncertainty for the dot-dashed line is represented by the shade. The dashed line represents the decaying behavior at $Q = 2.00$ Å^{-1} as discussed in the main text.
